# Supplementary material for: NET4 and RabG3 link actin to the tonoplast and facilitate cytoskeletal remodelling during stomatal immunity
Source: Nat Commun. 2023 Sep 20;14:5848. doi: 10.1038/s41467-023-41337-z (PMC10511709; doi:10.1038/s41467-023-41337-z)
Supplement: Supplementary file 1 — Supplementary Information [file 41467_2023_41337_MOESM1_ESM.pdf]

Supplementary Information

Hawkins and Kopischke *et al.* 2023

A NET4-RabG3 couple mediate the link between actin and the tonoplast and is essential for normal actin cytoskeletal remodelling in stomatal closure to flg22

Supplementary Figure 1. NET4 proteins bind actin and localize to the tonoplast.
Supplementary Figure 2. Characterization of *net4* mutant lines.
Supplementary Figure 3. Characterization of *rabg3b* mutants and complementation lines.
Supplementary Figure 4. NET4 co-localises with GTP-bound RabG3B at the tonoplast.
Supplementary Figure 5. Time course autophagic flux experiment for Col-0, *rabg3b* and *net4a/b* double mutant upon *flg22* treatment.
Supplementary Figure 6. Tonoplast morphology and vacuolar trafficking in *rabg3b* and *net4* mutants.
Supplementary Figure 7. Stomatal movements in Lifeact-mNeonGreen expressing Col-0 and *net4ab-c2*.
Supplementary Figure 8. Working model.
Supplementary Table 1. Potential NET4A interactors retrieved from tandem affinity purification (TAP)-tagging.
Supplementary Table 2. YFP-RABG3f interactors retrieved by immuno-precipitation from Arabidopsis.
Supplementary Table 3. Potential RABG3b interactors retrieved from yeast-two-hybrid screening.
Supplementary Table 4. Mutants used in this study
Supplementary Table 5. PCR primers used in this study for gene expression analysis and cloning constructs.

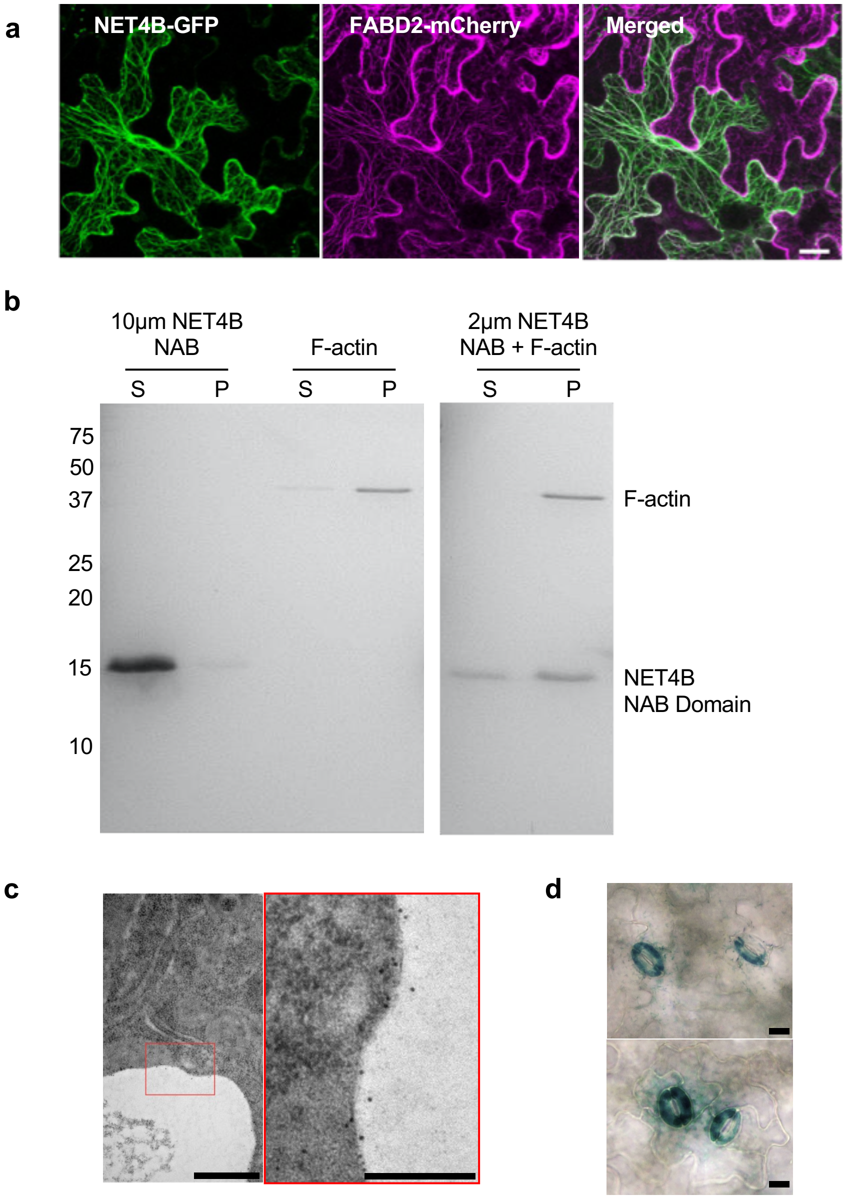

Supplementary Figure 1. NET4 proteins bind actin and localize to the tonoplast. **a** Confocal microscopy of *N. benthamiana* leaves transiently co-expressing NET4B-GFP and the actin marker mCherry-FABD2. Representative images are shown; scale bar = 20 µm. **b** Actin co-sedimentation assay. Recombinant NAB domains of NET4B (2µM) are mixed and co-sedimented with F-actin following ultra-centrifugation. **c** Transmission electron micrograph of anti-NET4B immunogold-labelled root sections. Gold particles are located in the vicinity of the tonoplast membrane. The tonoplast demonstrated the most abundant anti-NET4B labelling satisfying both criteria for preferential labelling giving a RLI (Relative Labelling Index) of 3.58, and a Chi squared 70.34% of the total. Scale bars = 500 nm and 200 nm (boxout). **d** GUS-stained mature leaves of the *NET4B* promoter- GUS reporter line. Scale bar = 20 µm. All experiments were repeated at least twice with similar results.

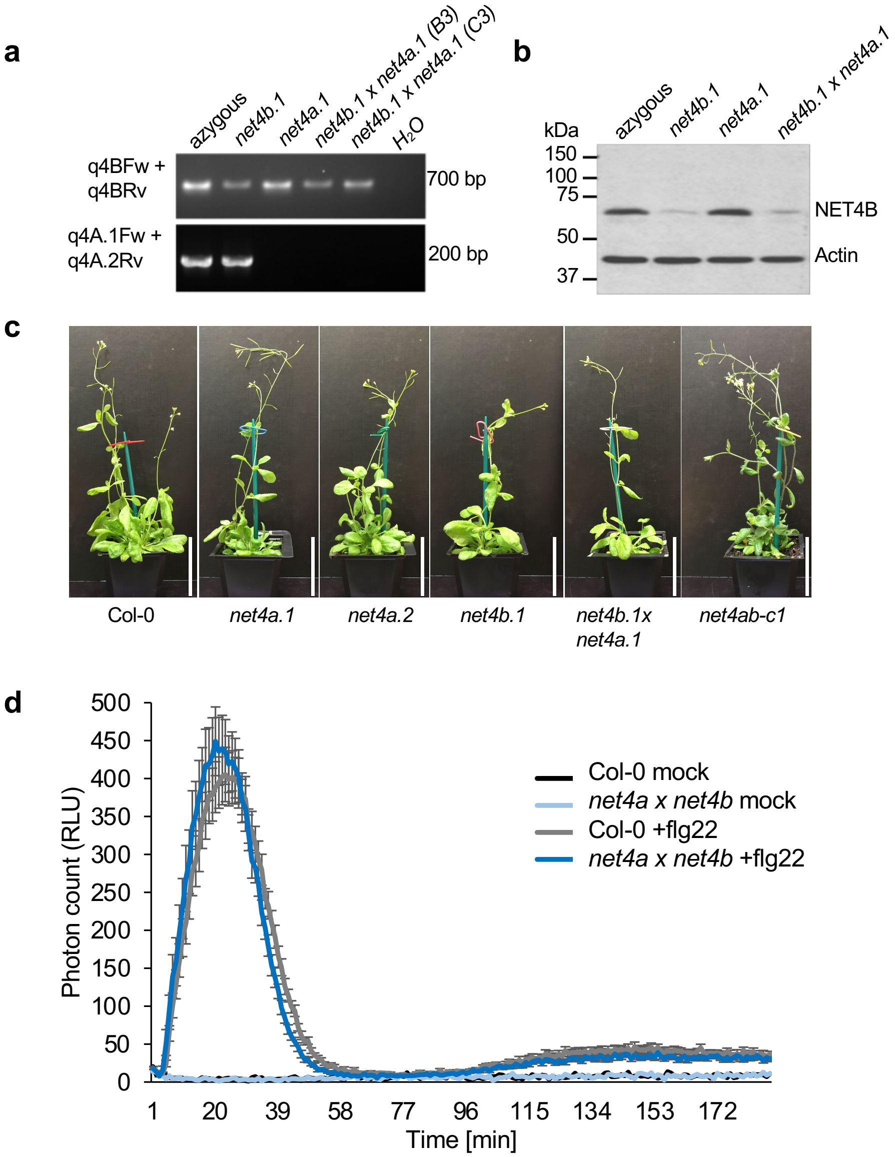

Supplementary Figure 2. Characterization of *net4* mutant lines. **a** Transcript levels of *NET4A* and *NET4B* upon their genetic disruption in homozygous *net4b.1*, *net4a.1* and *net4b.1 x net4a.1* T-DNA lines. No functional *NET4A* transcript is present in *net4a.1* and *net4b.1 x net4a.1*. However, *NET4B* transcripts are present in *net4b.1* and *net4b.1 x net4a.1* albeit at reduced levels, which represents a knock-down (kd). **b** Immunoblot analysis of total protein extracts from *net4b.1, net4a.1, net4b.1 x net4a.1*, and azygous plants using anti-NET4B antibodies. Blots show reduced NET4B protein levels in *net4b.1* and *net4b.1 x net4a.1*. **c** Macroscopic analysis of *net4* mutants indicate normal plant development and growth. **d** ROS production measured as relative luminescence units (RLU) in the indicated genotypes treated with 100 nM flg22 over time. Graph represents ±SEM; n = 12 leaf discs. Experiment is representative for two independent experiments with similar results.

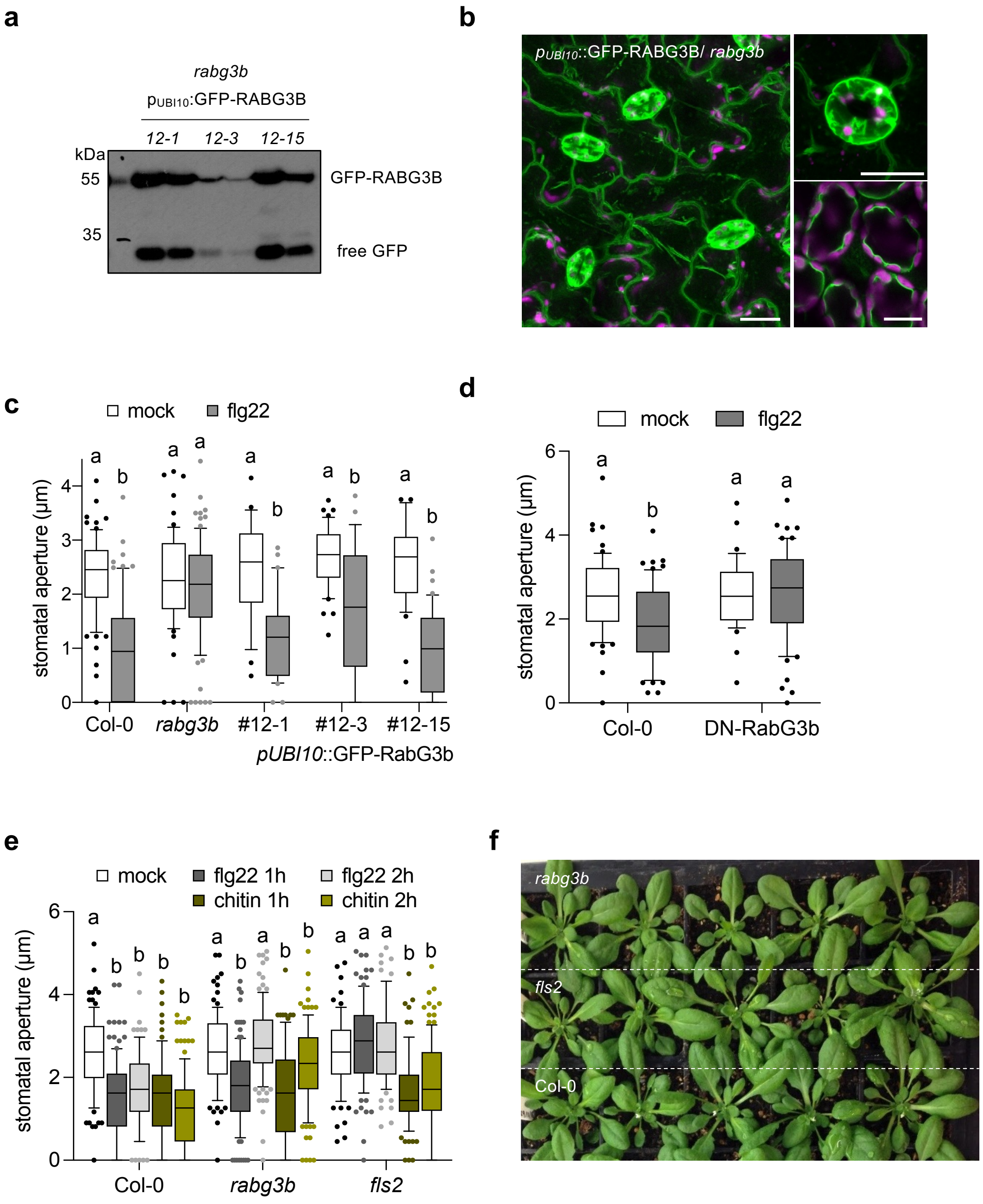

Supplementary Figure 3. Characterization of *rabg3b* mutants and complementation lines. **a** Immunoblot analysis of *rabg3b/*GFP-RabG3b expressing lines. Full-length GFP-RabG3b was detected using anti-GFP antibodies. The level of mutant complementation was correlated with the expression level of tagged RabG3b. **b** Confocal micrographs of *rabg3b/*GFP-RabG3b expressing lines. GFP fluorescence at the tonoplast could be observed in epidermal, guard and mesophyll cells. Shown are representative images overlaying signals from GFP fluorescence (green) and chlorophyll auto-fluorescence (magenta); scale bars = 20 µm. **c** Stomatal aperture measurements in the indicated GFP-RabG3b expressing genotypes

compared with Col-0 wild type and *rabg3b*. Stomatal apertures were measured 2 hr after treatment with 20 µM flg22. Box plots of the values are shown with whiskers from the 5th to 95th percentiles, the line in the box shows the median; Col-0 (mock) n = 76 stomata, Col-0 (flg22) n = 71, *rabg3b* (mock) n = 65, *rabg3b* (flg22) n = 87, #12-1 (mock) n = 21, #12-1 (flg22) n = 29, #12-3 (mock) n = 40, #12-3 (flg22) n = 40, #12-15 (mock) n = 37, #12-15 (flg22) n = 40 stomata. Different letters indicate significantly different values at p < 0.0001 (2-way ANOVA, multiple comparisons). **d** Stomatal aperture measurements in Col-0 wild type and plants expressing DN-RabG3b. Stomatal apertures were measured 2 hr after treatment with 20 µM flg22. Box plots of the values are shown with whiskers from the 5th to 95th percentiles, the line in the box shows the median; Col-0 (mock) n = 71 stomata, Col-0 (flg22) n = 65, line 75-9-9-7 (mock) n = 34, line 75-9-9-7 (flg22) n = 60 stomata. Different letters indicate significantly different values at p < 0.0001 (2-way ANOVA, multiple comparisons). **e** Stomatal aperture measurements in Col-0 wild type and *rabg3b*. Stomatal apertures were measured 2 hr after treatment with 20 µM flg22 (data shown in figure 5c) and 100 mg/ml chitin. Box plots of the values are shown with whiskers from the 5th to 95th percentiles, the line in the box shows the median; Col-0 (mock) n = 106 stomata, Col-0 (flg22 1h) n = 110, Col-0 (flg22 2h) n = 114, Col-0 (chitin 1h) n = 96, Col-0 (chitin 2h) n = 115, *rabg3b* (mock) n = 100, *rabg3b* (flg22 1h) n = 112, *rabg3b* (flg22 2h) n = 116, *rabg3b* (chitin 1h) n = 101, *rabg3b* (chitin 2h) n = 116, *fls2* (mock) n = 78, *fls2* (flg22 1h) n = 92, *fls2* (flg22 2h) n = 86, *fls2* (chitin 1h) n = 85, *fls2* (chitin 2h) n = 116 stomata. Different letters indicate significantly different values at p < 0.0001 (2-way ANOVA, multiple comparisons). The experiment was repeated at least twice with similar results. **f** Macroscopic analysis of *rabg3b* mutants indicate normal plant development and growth.

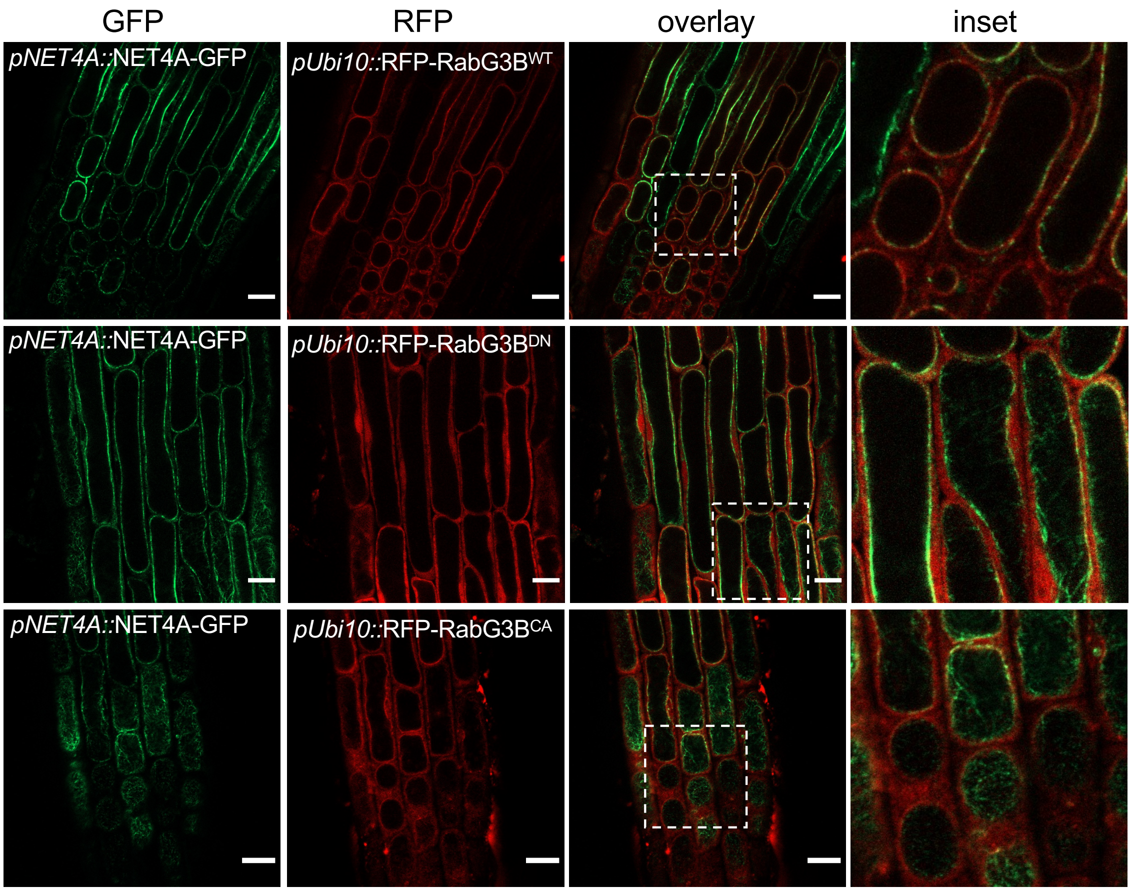

Supplementary Figure 4. NET4 co-localises with GTP-bound RabG3B at the tonoplast. Root tissues of stable transgenic Arabidopsis lines co-expressing *pNET4A::*NET4A-GFP with either *pUbi10::*RFP-RabG3B^{WT}, *pUbi10::*RFP-RabG3B^{DN}, or *pUbi10::*RFP-RabG3B^{CA}. RFP-RabG3B^{WT} localised to the tonoplast where it could be observed to co-localise with NET4A-GFP. RFP-RabG3B^{DN} appeared to exhibit a mostly cytosolic localisation pattern, and no obvious co-localisation with NET4A-GFP was observed at the tonoplast. RFP-RabG3B^{CA} localised to the tonoplast where it co-localised with NET4A-GFP. Scale bars = 10µm. These results were consistently observed in two independent experiments across which at least 11 plants were analysed.

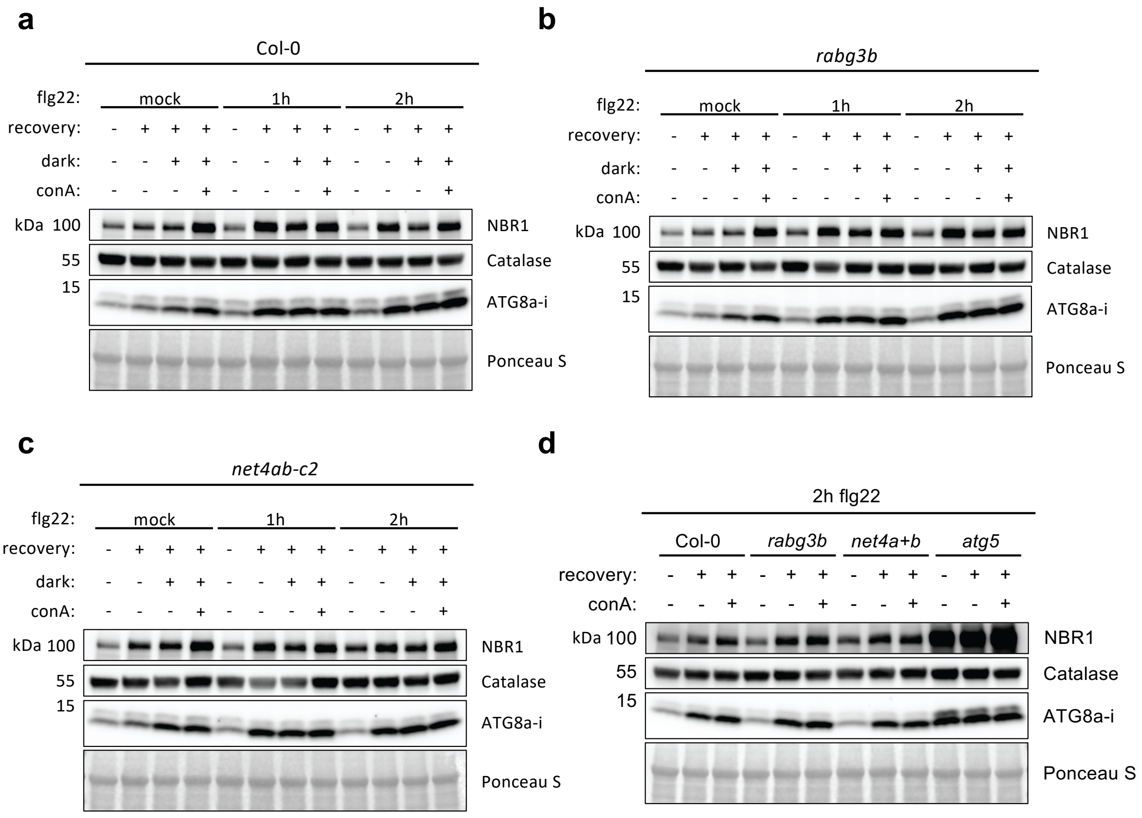

Supplementary Figure 5. Time course autophagic flux experiment for Col-0, *rabg3b* and *net4a/b* double mutant upon *flg22* treatment. Whole seedlings were treated with 10 µM *flg22* for the indicated time points and underwent a recovery period of 8h in (i) light, (ii) dark or (iii) dark and 1 µM conA. 15 µg of protein was loaded on each lane. Proteins were detected via anti-NBR1, anti-Catalase and anti-ATG8a-i antibodies, respectively.

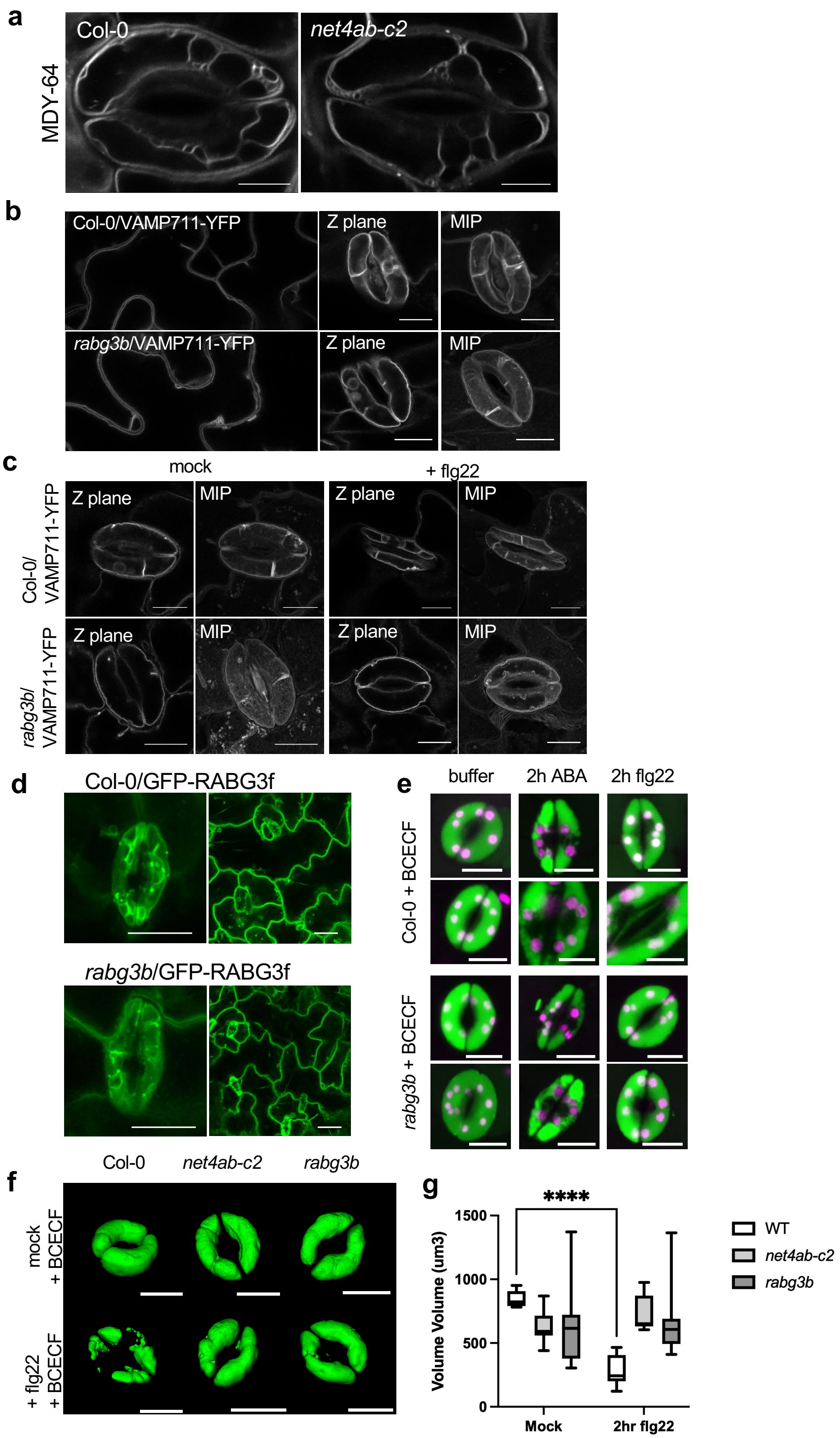

Supplementary Figure 6. Tonoplast morphology and vacuolar trafficking in *rabg3b* and *net4* mutants.

a Confocal micrographs of tonoplast membranes in *net4ab* mutants stained with the tonoplast-specific dye MDY-64. **b, c** Confocal micrographs of tonoplast membranes in transgenic *rabg3b* mutant lines expressing the tonoplast marker YFP-VAMP711. **b** Tonoplast localization of YFP-VAMP711 in *rabg3b* pavement and guard cells were similar to wild type (Col-0); scale bars = 10 µm. **c** Tonoplast localization of YFP-VAMP711 in *rabg3b* guard cells were similar to wild type (Col-0) whether open or upon closure induced by 20 µM flg22 for 2 hrs; scale bars = 10 µm (MIP = maximum intensity projection). **d** Confocal micrographs of vacuoles in leaf epidermis and guard cells of Col-0 (WT) and *rabg3b* seedlings visualised by constitutive expression of the tonoplast marker YFP-RABG3F; scale bars = 20 µm. **e** Vacuolar lumen staining with BCECF. The BCECF staining was performed after treatment with ABA (10uM) or flg22 (20 µM) to observe vacuolar re-arrangements during stomatal closure. **f, g** Confocal microscopy of Arabidopsis guard cell vacuoles of the indicated genotypes treated with 20 µM flg22. Vacuolar lumen was stained with BCECF. Scale bars = 10 µm. **f** Representative images of 3D vacuole reconstructions are shown. **g** Measurements of volumes from 3D vacuole reconstructions. Bars represent mean values ± SEM. WT Col-0 (mock) n = 11, *net4ab-c2* (mock) n = 10, *rabg3b* (mock) n = 11, WT Col-0 (flg22) n = 11, *net4ab-c2* (flg22) n = 11, *rabg3b* (flg22) n = 11 guard cells; ANOVA with Tukey's multiple comparisons test. Different letters indicate significantly different values at $p < 0.05$. The experiments were repeated at least thrice with similar results.

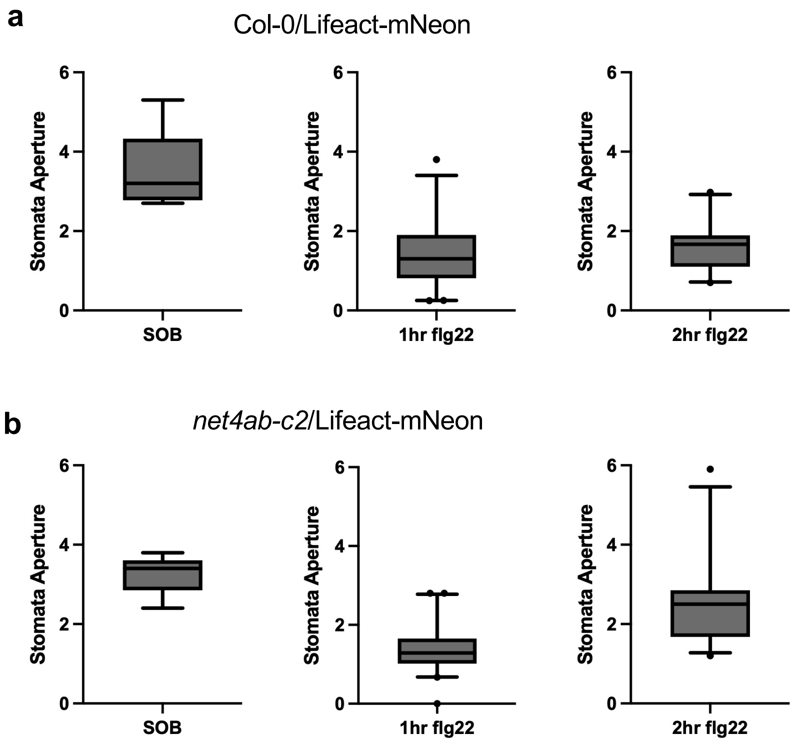

Supplementary Figure 7. Stomatal movements in Lifeact-mNeonGreen expressing Col-0 and *net4ab-c2*. **a, b** Stomatal aperture measurements of images used for quantitative evaluation of actin filament organization in guard cells from Col-0/Lifeact-mNeonGreen (WT) and *net4ab-c2*/Lifeact-mNeonGreen plants in stomata opening buffer (SOB) and after 1 and 2 hrs flg22 treatment. 1hr flg22 treatment: WT Col-0 n = 39, *net4ab-c2* n=44 guard cells; 2hr flg22 treatment: WT Col-0 n = 23, *net4ab-c2* n= 24 guard cells; Stomata opening buffer: WT Col-0 n=6, *net4ab-c2* n=6 guard cells. The experiments were repeated at least thrice with similar results.

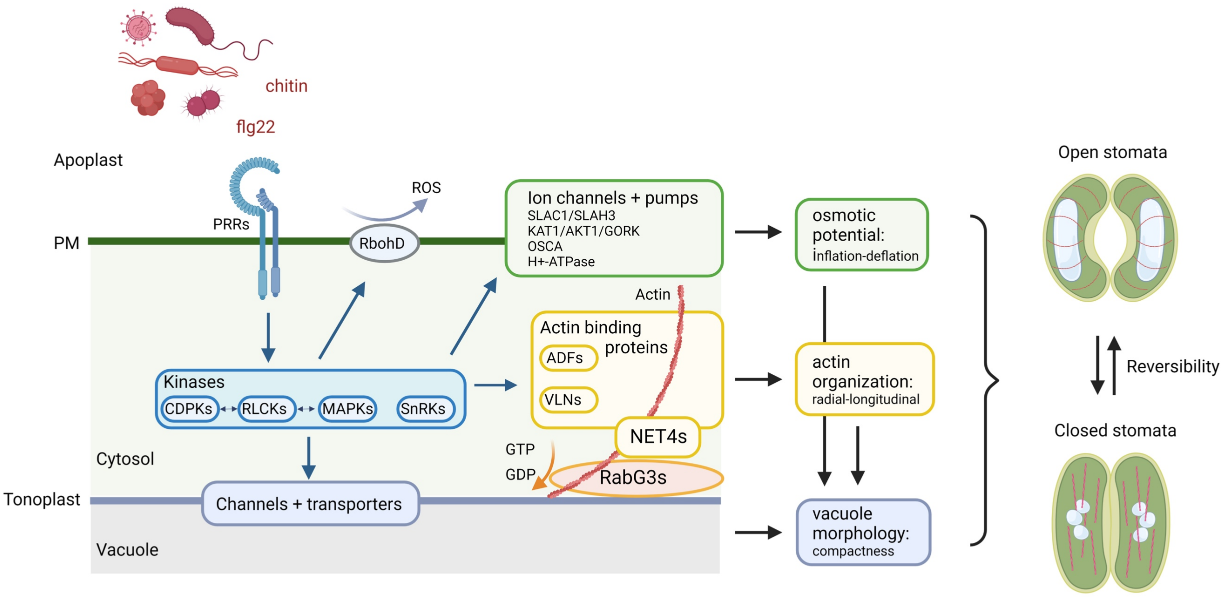

Supplementary Figure 8. Working model. Upon perception of PAMPs (flg22, chitin), signalling PRR complexes activate downstream kinases. These kinases i) activate ion channels, transporters and pumps at the plasma membrane and the tonoplast triggering ion fluxes, which change the osmotic potential of the guard cell and results in cell deflation accompanied by increasing the compactness of the vacuolar morphology; and ii) alter the activity of actin binding proteins, thereby changing the organization of the actin cytoskeleton from a radial to a more longitudinal array. The NET4-RabG3b couple tethers the reorganized actin filaments to tonoplast membranes, and our data suggest that this facilitates maintaining vacuole compactness of closed stomata. In mutants of the NET4-RabG3b couple, actin filaments can reorganize in response to PAMPs but guard cell vacuoles appear inflated, and thus, stomatal apertures are open. Since the mutants show initial stomatal closure and open stomata at 2 hrs PAMP treatment, we hypothesize that NET4-RabG3b actin-tonoplast tethering inhibits the re-opening of closed stomata. Figure was created with BioRender.

Supplementary Table 1. Potential NET4A interactors retrieved from tandem affinity purification (TAP)-tagging.

**ATG Code**		**1 % DDM**	**1% Digitonin**	**1% C12E8**	**1% TritonX100**	**Total**
AT5G58320	AtNET4A	+	+	+	+	4
AT4G09720	AtRABG3A		+	+	+	3

Supplementary Table 2. YFP-RABG3f interactors retrieved by immuno-precipitation from Arabidopsis. Co-immunoprecipitated proteins were quantified from at least three independent experiments and ranked according to their abundance in the YFP::RABG3f immunopreciptate relative to immunopreciptates with YFP::RABA2a and YFP::RABA5c and their abundance in total microsomes. The most highly ranked protein was AtVPS26A, a known interactor of RABG3f (Zelazny et al., 2013). NET4B consistently co-precipitated with YFP::RABG3f but never with YFP::RABA2a or YFP::RABA5c, nor was it detectable in total microsomes (P(plgem) = 0.005, 0.018, 0.028 respectively).

			Relative abundance*				Peptide detection stats					
Protein	ID	Description	RAB-G3f	RAB-A2a	RAB-A5c	Total microsomes	Length (AA)	mw (Da)	Indistinguishable Proteins	PSMs	Peptide Seqs	% Coverage
VPS26A	sp|Q9FJD0|VP26A_ARATH		50.9	0.00	0.00	0.00	302	35182	None	69	30	87
			24.40	0.00	0.00	0.00						
P(plgem)	NH=RAB-G3f			0.012	0.018	0.028						
NET4B	tr|Q84VY2|Q84VY2_ARATH	At2g30500 OS=Arabidopsis thaliana GN=At2g30500 PE=2 SV=1 NET4B	1.00	0.00	0.00	0.00	517	60287	None	10	7	16
SD			0.30	0.00	0.00	0.00						
P(plgem)	NH=RAB-G3f			0.005	0.018	0.028						
VPS35B	sp|F4I0P8|VP35B_ARATH	Vacuolar protein sorting-associated protein 35B OS=Arabidopsis thaliana GN=VPS35B PE=1 SV=1	69.12	0.35	0.00	0.06	790	89514	None	326	56	77
SD			12.42	0.61	0.00	0.12						

P(plgem)	NH=RAB-G3f			2.0E-05	1.9E-05	4.70E-05						
TRS120	tr|Q9FY61|Q9FY61_ARATH	Protein TRS120 OS=Arabidopsis thaliana GN=T5K6_30 PE=4 SV=1	0.56	7.81	6.47	0.31	1186	129712	None	281	62	61
SD			0.38	7.22	5.08	0.31						
P(plgem)	NH=RAB-G3f			1.58 E-03	3.44E -03	0.47						
	NH=RAB-A2a		1.58E-03		0.34	3.57E-04						
			n = 3	n = 5	n = 3	n = 6						

*SINQ quantification values all divided by 0.0000002759

Supplementary Table 3. Potential RABG3b interactors retrieved from yeast-two-hybrid screening.

ATG Code	Description	No. Clones
AT5G58320	AtNET4A	20
AT3G19900	Uncharacterised Protein	19
AT1G48540	Outer arm dynein light chain 1 protein	15
AT5G41790	CIP1 (COP1-INTERACTIVE PROTEIN 1)	7
AT2G25730	Uncharacterised Protein	6
AT2G14680	MATERNAL EFFECT EMBRYO ARREST 13	6

Supplementary Table 4. Mutants used in this study

**ATG number**	**Gene name**	**T-DNA line**
At4g09720	RABG3a	SALK_139519C
At2g22740	RABG3b	SALK_004938
At3g16100	RABG3c	SALK_021190C
At1g52280	RABG3d	GABI_967G07
At1g49300	RABG3e	SALK_058079C
At3g18820	RABG3f	SALK_122061C
At2G21880	RABG2	SALK_069603C
AT5G58320	NET4A	SAIL_116_C08
		SALK_083604
At2g30500	NET4B	SALK_056957

Supplementary Table 5. PCR primers used in this study for gene expression analysis and cloning constructs.

Gene name	ATG number	Forward primer 5’->3’	Reverse primer 5’->3’
U-Box	AT5G15400	TGCGCTGCCAGATAATACACTATT	TGCTGCCCAACATCAGGTT
RABG3A	At4g09720	CTTGATGAATCAATATGTGCATAA	CATCATAAACCAAAGCACAAC
RABG3B	AT1G22740	AATAGCCGAGTGGTATCTGAGA	AACCAGTATCTGGCTGGAAATAT
RABG3C	At3g16100	GAGTCGAGTGGTTACTGAGAA	ACTTCTTCTTCAGGTTCATTCT
RABG3D	At1g52280	TCTCAGAAACCACTCTGCTCT	GCTGGACAAGAAAGATTTCAA
RABG3E	At1g49300	ACTTTACAGATCTGGGACACA	GAAAGTTCTCTGGATCCGAAG
RABG3F	At3g18820	CTTATCGGAAACAAGGTTGAC	GAAAGCTTCCTCCACATTAGT
NET4A	AT5G58320	ATTATGATCTGCTTCGTTCCA	CTCCTCTATCAACTTCACCATC
NET4B	At2g30500	TAGCTTGATCATAACGCTCAG	AATCTAGAAAAGATGGATGATCG
net1b.1	At2g30500	CGGTGACGAGGCATTGATCCG	CTCGTCTTCGTGCATTCGCA
net1a.1	AT5G58320	ATGGATTATGATCTGCTTCGTTCCAAG AAG	GGTGGATGCCCTGAAAAGGC
EF1a Fw	AT2G18720	CCCATTTGTGCCAATCTCT	CACCGTTCCAATACCACCAA
TIP41	At4g34270	GTGAAAACTGTTGGAGAGAAGCAA	TCAACTGGATACCCTTTCGCA
mTAGBFP2		ccgggtaccATGGTCTCTAAAGGTGAGG	cttggcgcgccGTTGAGTTTGTGACCAA G
VAMP3		gcgccaagctatcaaacaagtttgtacATGAGTTT TCAAGATTTAGAATCAGG	gtttgaacgatcggggaaattcgagctcTCAAGC TGCGAGTACTATAATC
