# Supplementary material for: NET4 and RabG3 link actin to the tonoplast and facilitate cytoskeletal remodelling during stomatal immunity
Source: Nat Commun. 2023 Sep 20;14:5848. doi: 10.1038/s41467-023-41337-z (PMC10511709; doi:10.1038/s41467-023-41337-z)
Supplement: Supplementary file 2 — Description of additional supplementary files [file 41467_2023_41337_MOESM2_ESM.pdf]

Description of additional supplementary files

Supplementary Movie 1 : Confocal microscopy revealed discrete NET4A-GFP punctae labelling along the Lifeact-RFP actin filaments. Movie shows fly through of merged channel z series.

Supplementary Movie 2 : Arabidopsis lines co-expressing native promotor-driven NET4A-GFP and RABG3f-mCherry showed that NET4A localizes at the tonoplast Movie shows fly through of merged channel z series.

Supplementary Movie 3 : Arabidopsis lines co-expressing native promotor-driven NET4A-GFP and RABG3f-mCherry showed that NET4A localizes at the tonoplast. Movie shows a maximum intensity 3D render of a z series focusing on two vacuoles. First the NET4A-GFP signal is shown, then the RABG3f-mCherry signal, then a merge of the two channels.
